# Immune Thrombocytopenic Purpura: A Sequelae of Mycoplasma pneumoniae Infection

**DOI:** 10.7759/cureus.8955

**Published:** 2020-07-01

**Authors:** Pranay Bonagiri, Daniel Park, Joanna Ingebritsen, Deborah C Valtierra

**Affiliations:** 1 Internal Medicine, Touro University California, Vallejo, USA; 2 Family Medicine, Kaiser Permanente Vallejo Medical Center, Vallejo, USA; 3 Internal Medicine, Kaiser Permanente Vacaville Medical Center, Vacaville, USA

**Keywords:** mycoplasma pneumoniae, immune thrombocytopenia purpura

## Abstract

Our patient presented with a mild upper respiratory infection, which quickly developed into atypical pneumonia. Although atypical pneumonia is a common clinical presentation, our case is unique due to the rare hematologic complications associated with the underlying etiology of this atypical pneumonia case. Most cases of atypical pneumonia are simple, but ours developed immune thrombocytopenic purpura (ITP), which is a rare complication associated with an acute Mycoplasma* *pneumoniae infection (evidenced by positive IgM titers). Although our patient did not develop significant bleeding, our review of the literature demonstrated that ITP associated with M. pneumoniae infection can be fatal. Platelet count should be closely monitored and promptly treated.

## Introduction

Microbiology epidemiology is ever changing, but over the years Mycoplasma pneumoniae (M. pneumoniae) has remained steady as potentially the most common cause of atypical pneumonia with some sources even reporting it to be the cause of 35% of all community-acquired pneumonia (CAP) cases [1]. M. pneumoniae infection is associated with several extrapulmonary manifestations affecting almost every organ system. A rare extrapulmonary manifestation is thrombocytopenia, which is usually associated with thrombotic thrombocytopenic purpura (TTP) or disseminated intravascular coagulation (DIC). As opposed to the latter two, we present a case of immune thrombocytopenic purpura (ITP) associated with M. pneumoniae, which has been rarely reported in the literature. 

## Case presentation

A 46-year-old woman with a past medical history of anxiety disorder, allergic rhinitis, reactive airway disease, and prediabetes presented to the office for upper respiratory symptoms for one week. Her illness started with malaise, fatigue, and body aches. She proceeded to develop a dry cough with mild sinus symptoms. She reported a temperature of 100˚F for the last few days as well as shortness of breath during coughing episodes with minimal wheezing. She tried over-the-counter cough medications but to no avail. Upon examination, vital signs were normal with a blood pressure of 138/74 mmHg, a pulse of 88 beats/minute, a temperature of 99.7˚F, and an oxygen saturation of 99%. Pertinent positive findings included clear rhinorrhea. Pertinent negatives included clear lungs bilaterally with symmetrical air entry and no wheezes, rales, or rhonchi. She was diagnosed with an upper respiratory infection. Five days later, the patient had a telephone appointment and reported worsening productive cough with darker sputum, mild shortness of breath that improved with albuterol inhaler, and a max temperature of 102˚F. The clinician thought her symptoms were more suggestive of an upper respiratory infection with some reactive airway disease but could not fully exclude pneumonia. Outpatient labs were ordered. Based on these findings, the patient was asked to present to the hospital the next day. There she reported a headache, but no shortness of breath. She was on four liters of supplemental oxygen because of an oxygen saturation of 89% on room air when arriving at the emergency department. She did not have prolonged bleeding during blood draw or show other signs of bleeding. Upon physical examination, she was found to be borderline tachypneic and had bilateral diffuse crackles. 

**Figure 1:**
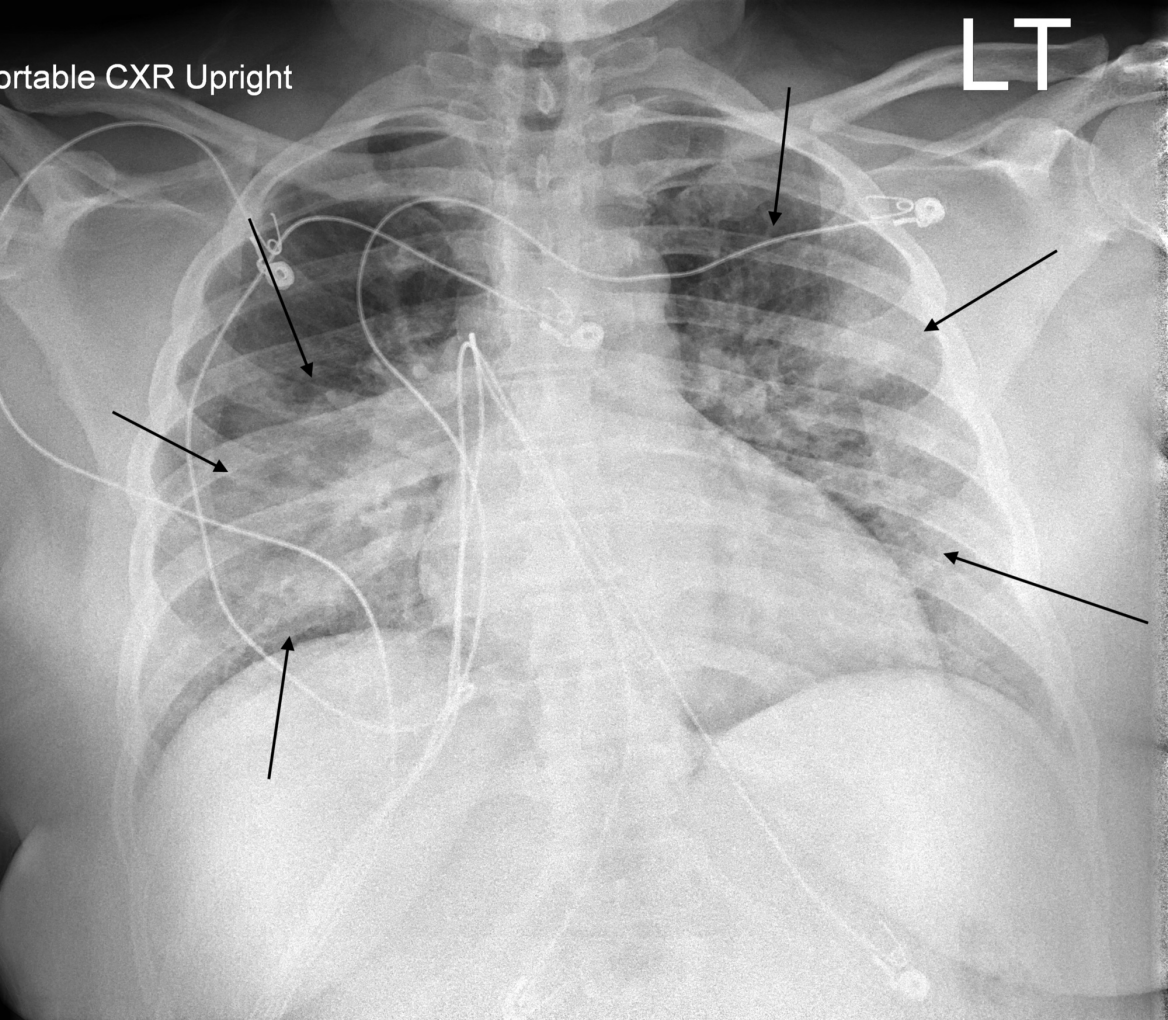
Anteroposterior chest x-ray demonstrating bilateral areas of consolidation consistent with atypical pneumonia

**Figure 2:**
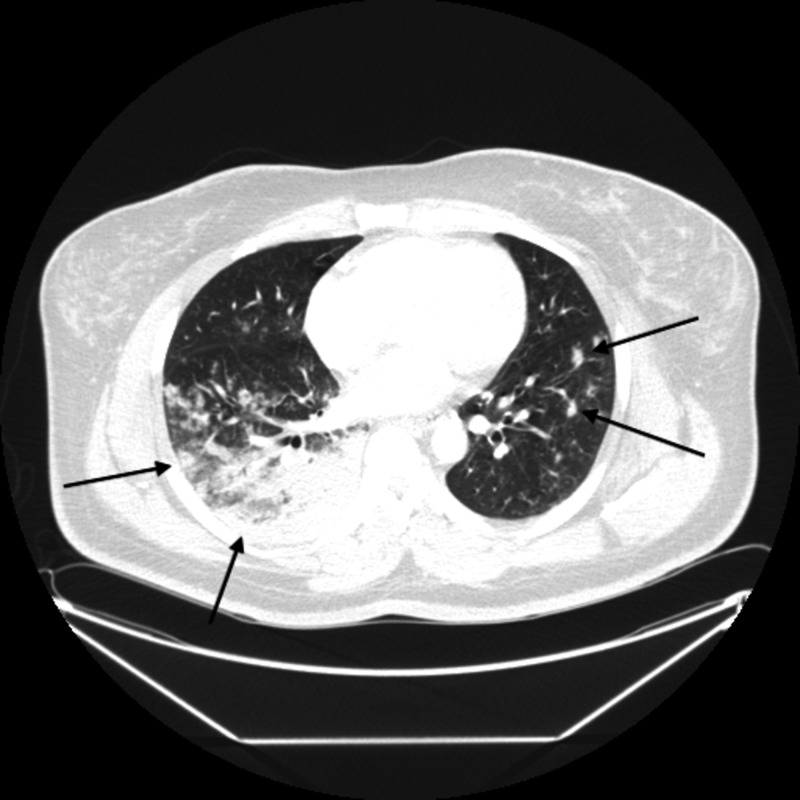
CT of the chest demonstrating bilateral consolidation, inflammation, and nodules more evident on the right side

The outpatient provider ordered procalcitonin, a complete blood count (CBC), and creatinine. When the results came back the following day, the patient was called in because of a platelet count of 4,000/µL. Her procalcitonin and creatinine levels were normal. On repeat CBC, platelets were found to be 1,000/µL with a hemoglobin of 10.2 g/dL. The white blood cell count was within normal limits. Her hemoglobin from the previous day was 11.3 g/dL. Her iron level was low at 27 µg/dL with a transferrin saturation of 11%. Her lactate dehydrogenase (LDH) was also mildly elevated (282 U/L). A chest x-ray suggested atypical pneumonia with findings of “bilateral areas of consolidation and pulmonary nodules concerning for multifocal infection or inflammation” (Figure 1). Additionally, CT of the chest was performed showing similar findings (Figure 2).

**Figure 3:**
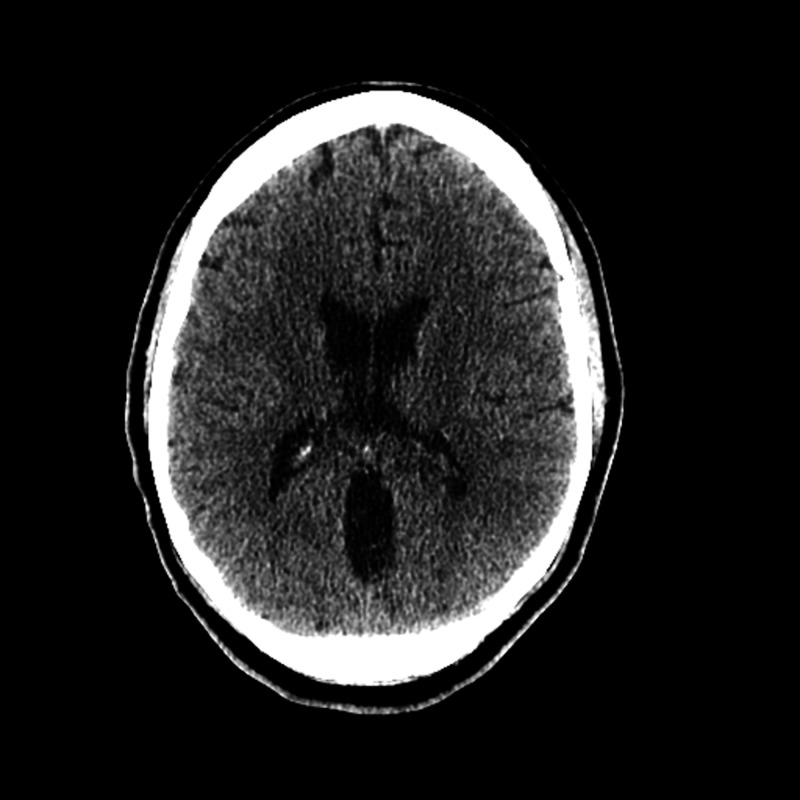
CT of head showing no evidence of intracranial pathology

A head CT was performed to rule out intracranial hemorrhage due to the severity of thrombocytopenia and found no evidence of acute intracranial process (Figure 3). Due to these lab findings, she was admitted to the hospital. The patient was also found to be positive for M. pneumoniae IgM, direct Coombs positive for IgG, and negative for complement. A peripheral blood smear was negative for schistocytes. 

The working diagnosis on initial presentation was viral upper respiratory infection, but this was refuted due to the worsening of symptoms on conservative measures. The final diagnosis of atypical community-acquired diagnosis due to M. pneumoniae was confirmed via chest x-ray at hospital admission and positive IgM. The secondary diagnosis of ITP was a diagnosis of exclusion. A normal peripheral blood smear lacking in schistocytes helped exclude TTP, DIC, and hemolytic uremic syndrome. The hemoglobin drop from 11.3 to 10.2 g/dL raised the suspicion of a hemolytic process, but after further investigation, it was more suggestive of iron deficiency anemia evidenced by a decreased iron serum level and decreased transferrin saturation. The hemoglobin corrected upon iron supplementation as well. TTP was less likely because ADAMTS13 was normal and LDH was not extremely elevated.

In the hospital, she was transfused one unit of platelets three times, started on two grams of intravenous (IV) ceftriaxone per day, 500 grams of IV azithromycin per day for five days, 1 mg/kg of ideal body weight intravenous immunoglobulin (IVIg), and 1 mg/kg/day prednisone. The IVIG and prednisone were started per hematology recommendation. The patient was also started on supplemental oxygen. The patient was discharged four days later on 120 mg per day of prednisone and 200 mg of cefpodoxime twice daily. The prednisone was tapered once platelet count was greater than 100,000/µL. On follow-up with hematology, dexamethasone 40 mg per day was prescribed for 10 days while prednisone was stopped. 

The patient was seen for follow-up three days after discharge. She was feeling better but reported a persistent mild cough. No positive findings were discovered on physical exam. Her platelets were now over 200,000/µL, and as she was finishing the course of cefpodoxime, a prednisone taper was started. Seventeen days later, the patient was seen for follow-up hematology consult. She reported residual cough, but otherwise was doing well. She did not report bleeding, bruising, petechiae, or headache. Follow-up labs three months later were normal as well.

## Discussion

ITP can be divided into two general groups: primary and secondary. Primary, or classic, ITP has become better understood in recent years. It seems to be due to IgG autoantibodies against GpIIb/IIIa or GpIb/IX/V in about 60% to 70% of patients [2]. Other less common causative factors include impaired megakaryocytopoiesis and T-cell-mediated destruction of platelets. On the contrary, ITP due to the M. pneumoniae infection belongs in the second group and this pathophysiology is less well understood. Potential mechanisms reported specifically for M. pneumoniae include platelet aggregation due to immune complexes, platelet clearance, and serotonin release [3]. Both Aviner et al. and Gouveia et al. reported cases and performed a literature report. The former reported seven cases of ITP, while the latter reported 10 cases though there was some overlap [3,4]. Although the exact mechanism of ITP is unknown, some hypotheses are less likely based on the data from the case series performed by Aviner et al. Contrary to classic ITP where antiplatelet antibodies are usually found, the authors included in the case series that did look for antiplatelet antibodies did not find any [4]. The mechanism involving immune complexes is less likely based on this finding. Another suggested mechanism is the direct interaction between the M. pneumoniae organism and the platelet, which would lead to the recognition of the platelet as foreign by the immune system [4].

One big difference between ITP associated with M. pneumoniae and ITP associated with other infections is the time course. Classically, ITP occurs days to weeks after an infection, but in our case and the others reported by Aviner et al. and Gouveia et al. ITP occurred alongside with the M. pneumoniae infection. The severity of the thrombocytopenia and the clinical manifestation differed among the cases reported by Aviner et al. and Gouveia et al. Between the two, platelet count on presentation ranged from 2,000 to 66,000/µL. However, the symptoms and signs associated with the platelet count do not seem to be very well correlated. On presentation, our case had a platelet count on the lower end of the spectrum (4,000/µL) and was completely asymptomatic. Another case with the same presenting platelet level developed purpura, epistaxis, and hematuria [5]. A patient with a higher platelet count on presentation (7,000/µL) reported much more severe symptoms including a brain stem hemorrhage, which led to his death [4].

## Conclusions

Although a rare manifestation of M. pneumoniae infection, ITP can be potentially life threatening. As discussed above, there is a wide array of clinical presentations and the worse thrombocytopenia should not be associated with worse outcomes. Based on this, the clinician should be cognizant of even mild thrombocytopenia to prevent complications. Further research needs to be performed to determine the exact mechanism of ITP secondary to M. pneumoniae infection, but this relationship is important because of the ubiquity of M. pneumoniae infections in the United States. Clinicians should be aware of this relationship, so they can provide empiric treatment specific for M. pneumoniae infection before serology returns to confirm the infection. 

## References

[1] Parrott GL, Kinjo T, Fujita J (2016). A compendium for Mycoplasma pneumoniae. Front Microbiol.

[2] Lambert MP, Gernsheimer TB (2017). Clinical updates in adult immune thrombocytopenia. Blood.

[3] Gouveia C, Evangelista V, Almeida R, Baptista AM (2018). Immune thrombocytopenia associated with Mycoplasma pneumoniae infection. Eur J Case Rep Intern Med.

[4] Aviner S, Miskin H, London D, Horowitz S, Schlesinger M (2011). Mycoplasma pneumonia Infection: a possible trigger for immune thrombocytopenia. Indian J Hematol Blood Transfus.

[5] Beattie RM (1993). Mycoplasma and thrombocytopenia. Arch Dis Child.

